# Integration of virtual platforms for enhanced conference experience: Data-based evidence from the Society of Interdisciplinary Placebo Studies 2021 conference

**DOI:** 10.3389/fcomm.2022.857661

**Published:** 2022-08-18

**Authors:** Jessica Cornell, Ariana Taj, John Sivinski, Margaret Yin, Parth Bhatia, Desai Oula, Sophia Fatschel, Patricia Franklin, Jason Noel, Luana Colloca, Chamindi Seneviratne

**Affiliations:** 1 Department of Psychiatry, University of Maryland School of Medicine, Baltimore, MD, United States,; 2 Department of Pain and Translational Symptom Science, University of Maryland School of Nursing, Baltimore, MD, United States,; 3 Institute for Genome Sciences, University of Maryland School of Medicine, Baltimore, MD, United States,; 4 Office of Research Administration, MPowering the State Initiative University of Maryland, College Park, MD, United States,; 5 College of Arts and Sciences, Ohio State University, Columbus, OH, United States,; 6 Depatment of Pharmacy Practice and Science University of Maryland School of Pharmacy, Baltimore, MD, United States,; 7 Center to Advance Chronic Pain Research University of Maryland, Baltimore, MD, United States

**Keywords:** virtual conference, SIPS, placebo, expectations, alcohol, pain, addiction

## Abstract

**Background:**

The Society of Interdisciplinary Placebo Studies (SIPS) was one of many organizations that hosted a virtual scientific conference in response to the COVID-19 pandemic restrictions. Retaining essential benefits of an in-person conference experience was a primary objective for the SIPS conference planning committee and guided the selection of a virtual platform on which to host the 2021 meeting. This article reports on the methods used to design and analyze an engaging, virtual scientific conference, along with the findings and implications for future meetings.

**Methods:**

Participant use of and interaction with different features of the conference platform were recorded and exported for analysis. Additionally, all SIPS conference attendees were invited to complete a brief, online post-conference survey that inquired about their perceptions of the SIPS conference specifically as well as their opinions of virtual and hybrid conferences in general. Using these data, we assessed (1) attendance patterns, (2) level of engagement, and (3) attendee satisfaction.

**Results:**

The platform recorded 438 unique, active conference attendees who used either a mobile app, web browser, or both to participate during the 3-day program. Seventy-four percent (*N* = 324) of active users attended all 3 days with 30 and 26 new attendees on Days 2 and 3, respectively. The *connections* feature offered on the platform was the most utilized function within the online forum. Attendance in the parallel workshop sessions remained constant across the 3 days, with an average of 44.6% (SD = 6.77) of people moving between workshops within a single session. The two poster sessions had an average of 47.6 (SD = 17.97) and 27.8 (SD = 10.24) unique views per poster, respectively. Eleven percent (*N* = 48) of attendees completed the post-conference survey. Thirty-six percent of these responders stated they were only able to attend because the conference was offered virtually. Further, the quality of the conference had an average satisfaction rating of 68.08 out of 100 (SD = 22.94).

**Conclusion:**

Results of data analyses suggest the virtual platform allowed for those who were unable to attend to join virtually, produced moderate engagement throughout the conference, and that the majority of attendees were satisfied with the quality of the fully-virtual conference. Therefore, incorporating virtual aspects in future in-person conferences could enhance conference experience and participation.

## Introduction

Many scientific conferences made the transition from in-person to entirely virtual events in line with recommendations published in response to the COVID-19 pandemic (Bosslet et al., 2020; Kopec and Stolbach, 2020; Lazaro et al., 2020; McDowell et al., 2020; Rundle et al., 2020; Rush et al., 2020). Often, organizations had less than a week to transition their on-site conferences to a virtual format (Bosslet et al., 2020; Fulcher et al., 2020; Kopec and Stolbach, 2020; McDowell et al., 2020).While the transition to a virtual platform presented challenges for organizers, benefits were also observed. Specifically, conference organizers reported increased attendance at virtual conferences compared to previous registration numbers at on-site programs. Virtual conferences became more accessible at one level due to the reduced costs (no travel, lodging, or food) and eliminated time needed for travel. Certain features of virtual conferences (e.g., polling and Q&A) allowed for increased audience engagement while facilitating a comfortable environment that encouraged those who would not normally speak during networking sessions to do so (Bosslet et al., 2020; Kopec and Stolbach, 2020; Rotoli et al., 2020; Aravamuthan et al., 2021).

In contrast to the numerous benefits, technological difficulties were one of the main challenges experienced: individual microphone access, sound optimization, and general connectivity issues impeded conference flow (Rundle et al., 2020). Another major difficulty reported was the limited networking capabilities in virtual formats. The organic networking experience of in-person conferences connected individuals and spurred novel scientific ventures (Hauss, 2020). Repeated findings indicated that networking tended to be less successful on virtual platforms (Kopec and Stolbach, 2020; Aravamuthan et al., 2021). The organized structure of virtual networking may even make it difficult for an additional party to naturally join an ongoing discussion (Aravamuthan et al., 2021). Without spontaneous interactions as an impetus for conversation, virtual networking seemed to be less attractive to regular attendees of these conferences (Bosslet et al., 2020; Fulcher et al., 2020; Kopec and Stolbach, 2020). However, other studies on virtual networking within conferences found that greater structure can make virtual networking as, or more fulfilling than the traditional networking experiences, especially for students (Fulcher et al., 2020; Aravamuthan et al., 2021). Overall, the literature provided new insights into designing virtual conferences.

However, it is important to note that only a few studies included robust quantitative data on participation, networking, and other elements of the attendee and speaker experience (McDowell et al., 2020; Stein et al., 2021). In addition, the popularity of virtual environments demands further investigation on their application to virtual scientific conferences. Potential benefits to post-COVID era conferences have also not been thoroughly explored.

Therefore, as a result of the 3rd International Conference of the Society for Interdisciplinary Placebo Studies (SIPS) pivoting to a fully, virtual platform, the potential to collect extensive quantitative and qualitative metrics leading up to, during, and after the conference provided an opportunity to further explore the impact of a virtual scientific meeting.

The following presents the design, transition to, and implementation of a virtual platform at the 2021 SIPS conference. We also address gaps in the literature and discuss implications of the data we have collected pre-, during, and post-conference that may benefit future conferences seeking to integrate aspects of in-person and virtual platforms for improved experience.

## Methods

### Organization

A formal Conference Planning Committee was established in March 2018, comprising of four faculty members from University of Maryland, Baltimore (UMB). In February 2021, committee members invited seven UMB-affiliated students to participate as volunteer support for the conference. As a result of the COVID-19 pandemic and closure of the UMB campus, one volunteer was based in India, and another based in Ohio during the entirety of conference planning and execution.

During committee meetings, the conference agenda was developed, focusing on (1) expanding the scope of topics beyond that of previous SIPS conferences, (2) providing historic perspective as well as the current state of the science, (3) facilitating translation of the science to practice, (4) engaging senior as well as emerging investigators in the field, and (5) providing a friendly forum for professional networking. The conference design included seven plenary sessions, a lifetime achievement lecture, three special sessions, including a timely conversation panel on COVID-19, and three career development sessions. The goals and themes of the conference were also fulfilled through the 21 parallel workshops, two poster sessions (49 presentations), and five oral presentation sessions consisting of 54 presentations. The program offered multiple forums provided opportunities for senior, mid-level, and early-career level researchers, and practitioners to give thoughtful presentations on their respective scientific research, utilizing several forms of media.

### Response to COVID-19 pandemic

The Planning Committee closely monitored national and international developments, along with UMB policies and international recommendations related to the COVID-19 pandemic, which became a standing agenda item on the weekly Committee meetings. The decision to shift from in-person to a virtual meeting was decided in November 2020. Once this decision was made, funds initially dedicated to support an in-person meeting were reallocated to support a robust virtual platform. A search was conducted for a platform that offered the following elements within the available budget:

- Supported live and pre-recorded presentations
- Included proven user-friendly navigation
- Provided multiple mechanisms to enhance participation
- Facilitated real-time interactions between attendees and speakers
- Provided forums for networking
- Allowed customization of online platform
- Provided technical support before, during, and after conference
- Provided data of conference participation

Committee members interviewed company representatives and requested proposals and quotes from potential vendors. *SOCIO Inc*. (Indianapolis, IN, USA; now part of Webex) was selected as the company that best met the platform and budget criteria (https://SOCIO.events/aboutus). The Planning Committee worked with SOCIO staff to custom develop a visually appealing and engaging virtual conference site. Customizing the platform was a lengthy process that continued non-stop up to the start of the event. Adjustments were also made throughout the 3-day conference. For additional details on the SOCIO features used, please see Table 1.

### Conference operations

Technical support is crucial to all meetings, yet virtual platforms impose additional technical challenges for both speakers and the audience. While SIPS speakers received detailed written instructions and opportunity to practice in the platform prior to the conference, one to two Committee members were assigned as “Tech Support” (TS) for each session to assure reliable technical support during their Conference presentation. Parallel sessions with multiple, simultaneous presentations, had an additional Committee member serve as a monitor for the entire period. The TS had multiple responsibilities: ~20 min before the start of a session, TS met with speakers in the pre-assigned livestream room to review the room’s features as well as check that all audio-visual pieces to the presentation were operating. Once all were ready, the TS would start the livestream. A private chat function allowed speakers and the TS to communicate separately from the audience (e.g., “You will be going live in 5 min.” or “Is my screen still sharing?”). The TS would also use an audience chat feature to communicate any issues and check for technology problems (e.g., sound quality, video quality, and lag), as well as prompt and moderate audience participation during the session.

As part of the commitment to excellence, the entire Planning Committee met at the close of each conference day and conducted a debriefing of the day’s proceedings. These meetings identified issues to be addressed by the SOCIO staff, shared strategies for managing common issues encountered during live sessions, developed communications to update Conference attendees, as well as anticipated needs for the next day in order to mitigate any problems.

### Attendee experience

One week prior to the conference, all registrants received a link to the SOCIO platform and encouraged to develop a personal account and profile, become familiar with the features of the platform as well as review the agenda. Links to individual sessions were not activated until the conference day they were scheduled. Attendees were able to view each speakers’ biography and related sessions. In addition, they could view information about other attendees and had the ability to form a virtual *connection* (virtual private interaction) by sending an invitation and having the invitation be accepted, similar to “friending” someone on social media. Once a *connection* was made, two people could start a conversation.

To join a session, attendees navigated to the “Agenda” tab where all sessions were listed by date and time (user’s local time zone), then clicked on an agenda item or the “Join Livestream” button below each session. During live sessions, attendees used the chat function to send comments and questions to speaker(s) as well as to other attendees. The TS would monitor the chat and share questions with the speaker(s). Due to limited livestreaming room availability, if the Q&A part of the session ran past its scheduled time, attendees and speaker(s) were then directed to smaller breakout rooms to continue the discussion.

### Q&A rooms

Following each live session, attendees with unanswered questions were asked to move to a specific Q&A room assigned to that session. In these rooms, attendees could turn on their cameras and engage in a live conversation with the speaker. These rooms had a capacity for 16 attendees including the three reserved spots for conference staff and speakers. The session TS would also accompany speaker and monitor the room so that anyone who wanted to participate, had an opportunity.

### Networking

Dedicated times for social engagement such as networking breakfasts, lunches, and social events were interspersed throughout the conference and were open to all attendees and speakers. Dedicated networking rooms were also available 24/7, each with the capacity for hosting 16 people including reserved spots for Committee members and speakers. Attendees were able to use these networking rooms at any point during the conference. Discussions could also be conducted in the “connections” feature where attendees had the option to privately chat with one or more attendee at a time.

The entire program provided multiple avenues for supporting networking. As mentioned earlier, attendees were also able to interact with speakers and other attendees using a chat function during plenary sessions, spotlight sessions, oral presentations, and in the Q&A rooms. Poster presentations provided a forum to discuss and network with presenters and other attendees through face-to-face video or through the chat function.

### Post-conference

#### Sustaining efforts

Each plenary, workshop, and spotlight session were recorded and saved to the SOCIO platform. The videos were then edited to minimize errant audio or visual issues. A link to the recorded sessions was posted on the SOCIO platform under the specific session. All registered participants were then able to view these videos. In addition, these recordings were used to develop an “on-demand conference” for those who were unable to attend the live event and sustain the impact of this program.

#### Survey development

A conference assessment survey was developed using the program REDCap, a HIPAA compliant web application for data capturing and storage, for the purpose of understanding their experience of the 2021 SIPS virtual conference. The survey (Supplementary material) was designed to elicit participants’ perceptions both specific to the virtual format as well as how they compared the virtual conference to in-person conference experiences. To maximize survey participation, the survey was designed so that it could be completed within ~10 min. Briefly, the information gathered from the survey participants included participant background information, conference experience (on a scale from 0 to 100), and plans for future conference participation.

The survey was reviewed by the entire SIPS Conference Planning Committee and submitted to UMB’s Institutional Review Board (IRB). After receiving exempt status by the IRB, the survey was sent out by email ~2 months after the close of the conference. Participants accessed the survey by clicking a link that opened the survey in a separate browser. A survey disclosure statement was displayed prior to the start of the survey. The survey was voluntary and anonymous, with consent being explicit through agreement of participation.

#### Collection of data on attendee conference activity

Individual attendee conference activity, including *connections* made, attendance for each session, poster and poster external link views and networking and Q&A room attendance was recorded real-time in individual logs on the SOCIO platform. Each attendee who registered for the conference and created an account on the SOCIO platform was identified as “active”. After the conference, activity logs for all active attendees were downloaded from SOCIO and combined into a single master file of de-identified data used for analysis in Microsoft Excel. SOCIO employees and support staff activity data were excluded from analysis. Those who registered for the conference but had not created an account were considered as “active” attendees, and therefore were not included in the analyses.

### Statistical analysis

To compare the average attendance per session across days, we performed a Levene’s test to check if variance was statistically significantly unequal across the 3 days, and a one-way ANOVA test was performed to determine if the average attendance significantly differed across the 3 days. All statistical analyses were performed using the software package R (The R Foundation, Vienna, Austria).

## Results

### Attendance levels

**FIGURE 1:**
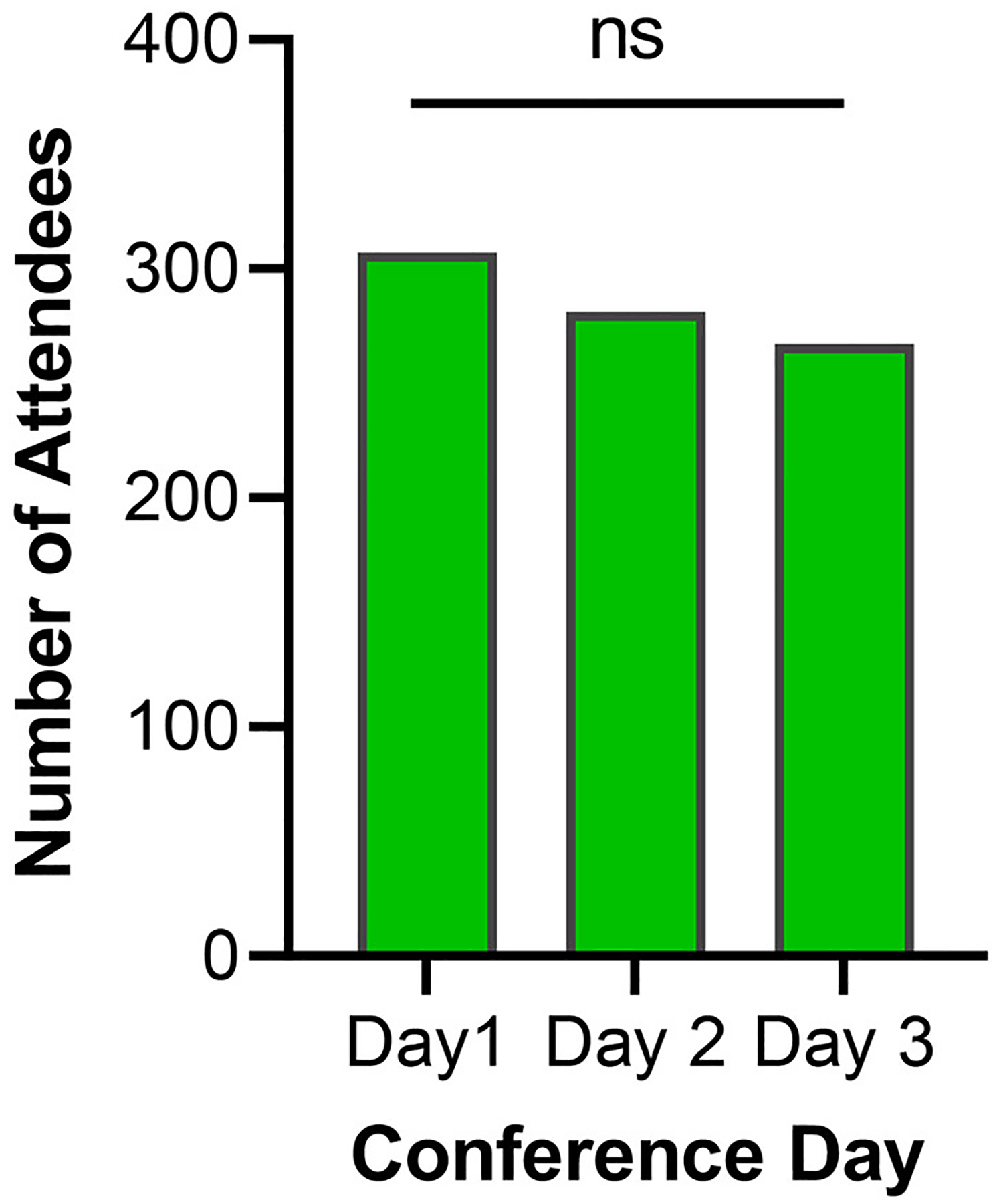
Number of attendees across the three conference days. ns, not significant represents a *p*-value > 0.05. Statistical analysis by Chi square goodness-of-fit test, *p* = 0.2477, χ^2^ = 2.791.

The SOCIO platform counted 438 active users from 20 different countries across the 3 days of the conference (Table 2). Attendance was measured as activity originating from either a web browser (77%) or the SOCIO mobile app (23%). Seventy-four percent of active users attended all 3 days, with a slight decline in total attendance observed on each subsequent day. Additionally, 30 and 26 new attendees joined on Day 2 and 3, respectively. No significant difference in attendance across the 3 days (*p*-value = 0.2477) was observed (Figure 1). Furthermore, average attendance across 3 days showed no significant unequal variance (*p*-value = 0.79). Ten percent (*N* = 48) of attendees completed the post-conference survey. Of those who completed the survey, 36% stated they were only able to attend because the conference was offered virtually.

### Conference activity levels

**FIGURE 2:**
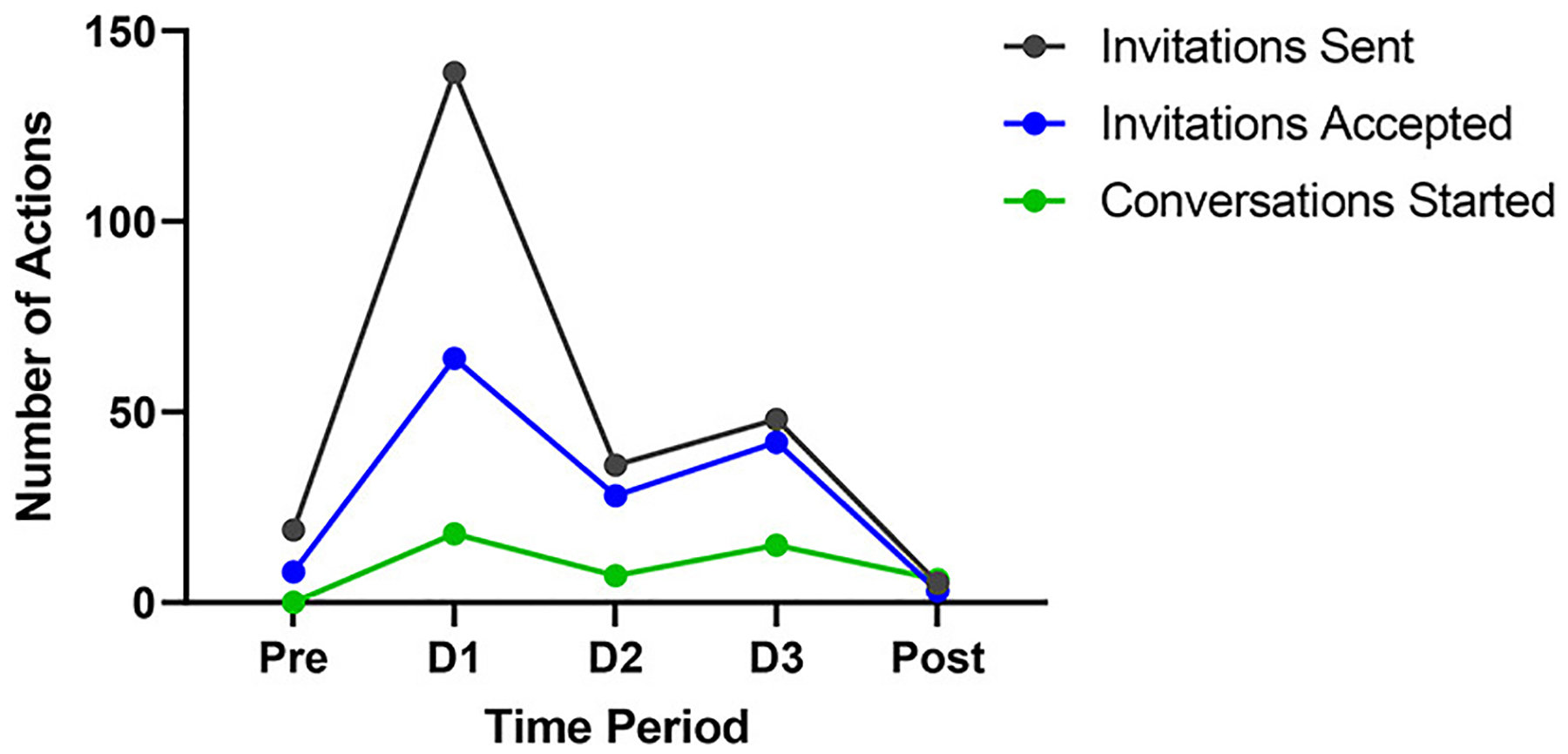
Connections made and conversation started from pre- to post-conference. Invitations sent and invitations accepted (*connections* made) from pre-conference to post-conference. Conversations started for Pre-conference (Pre), Day 1 through 3 of the Conference (D1, D2, and D3), and Post-conference (Post).

Conference activity levels were measured by networking room utilization rate, session and poster attendance, and the number of *connections* and conversations recorded. A total of 89 (20.3%) unique users made use of a networking room across all 3 days with 55, 49, and 12 unique users recorded on Day 1, 2, and 3, respectively. The number of attendees who participated in parallel workshop sessions did not significantly differ across 3 days (Levene’s test: *p* = 0.5; ANOVA: *p* = 0.273), with 44.6% of people moving between workshops within a session, whereas the educational sessions (*n* = 3) had 26% (*N* = 27) of people moving between sessions. Figure 2 shows that *connections* (invitations sent and accepted) and conversations varied from pre- to post-conference duration, where pre-conference pertains to days leading up to the Conference since the activation of the SOCIO platform, and post-conference pertains to the period starting after Day 3 of the Conference. Of the 247 invitations sent throughout the conference, 59% were accepted and 19% of the invitations sent resulted in conversations.

**FIGURE 3:**
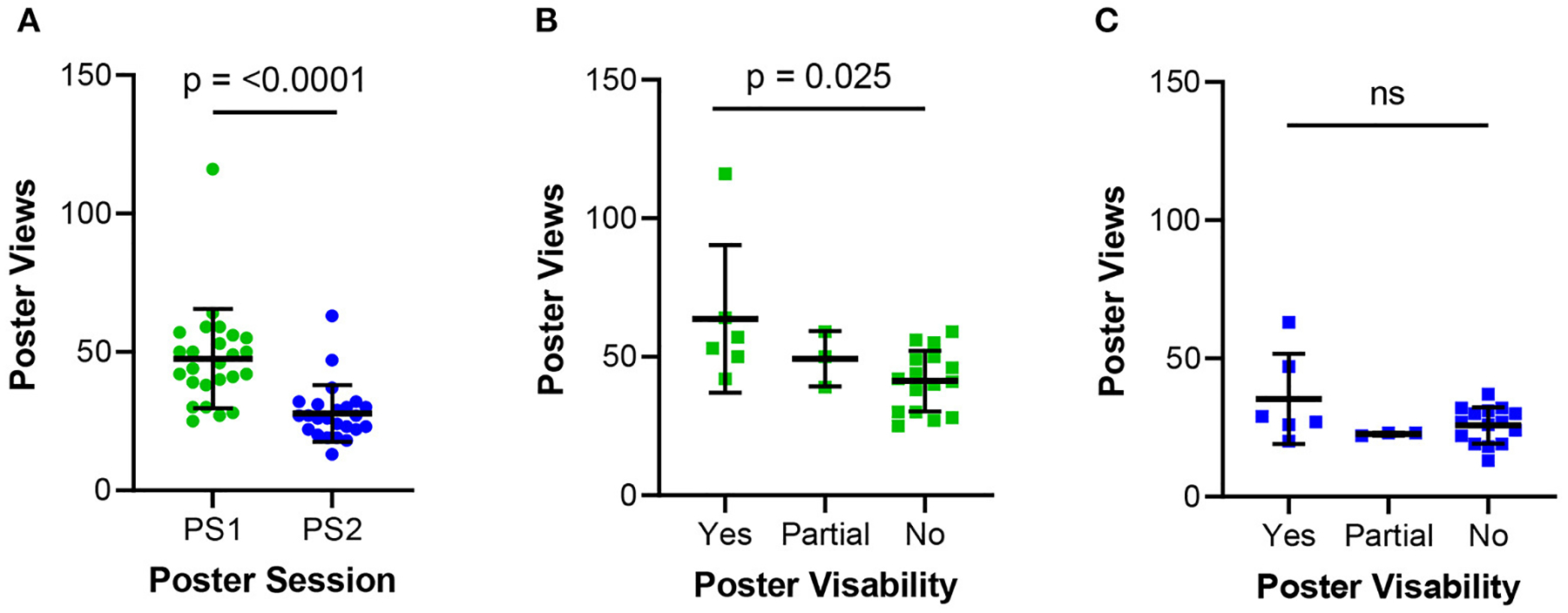
Poster views during the two sessions. **(A)** Poster session views per poster for Poster Session 1 (PS1) and Poster Session 2 (PS2) on conference Day 1 and 2, respectively. **(B)** Poster views separated by initial visibility when navigating to the Poster Session features for PS1 and **(C)** PS2. Error bars represent standard errors of mean. Statistical analysis by Levene’s test and ANOVA [Poster Session 1: Levene’s test (*p*-value = 0.476), one-way ANOVA (*p*-value = 0.025); Poster Session 2: Levene’s test (*p*-value = 0.121), one-way ANOVA (*p*-value = 0.09)]. ns, not significant represents a *p*-value > 0.05.

Posters presented in Session 1 each received 47.6 (SD = 17.97) unique views while posters in Session 2 each had 27.8 (SD = 10.24) unique views (Figure 3A). A significant difference was observed in views per poster, according to placement of poster on the website for Session 1 but not for Session 2 [Poster Session 1: Levene’s test (*p*-value = 0.476), one-way ANOVA (*p*-value = 0.025); Poster Session 2: Levene’s test (*p*-value = 0.121), one-way ANOVA (*p*-value = 0.09)] (Figures 3B,C). Of the 49 posters, 38 posters contained an external link to either an audio file or video file of their poster for a total of 220 view with a mean of 6 (SD = 4.79) views per external link.

### Post-conference survey

The post-conference survey allowed attendees to provide feedback on their experience with the SIPS conference. Fifty-nine attendees began the post-conference survey however 11 surveys were excluded from the analysis due to incompleteness. The resulting 48 completed surveys used in this analysis represent 11% of the total conference attendees, which was an insufficient number of responders to assure validity.

Responders had the option to select multiple academic discipline and career stage categories. Results of the survey indicated approximately half of the survey responders represented psychology (47.9%, *n* = 23) and career stage of survey responders was distributed between early-career (31.3%, *n* = 15), mid-career (22.999%, *n* = 11), and senior-level career (35.4%, *n* = 17) investigators. Over half of survey responders were between the ages of 25 and 44 (56.25%, *n* = 27). Lastly, 95.83% (*n* = 44) of responders attended the conference were located in either North America or Western Europe (Supplementary Table 1).

**FIGURE 4:**
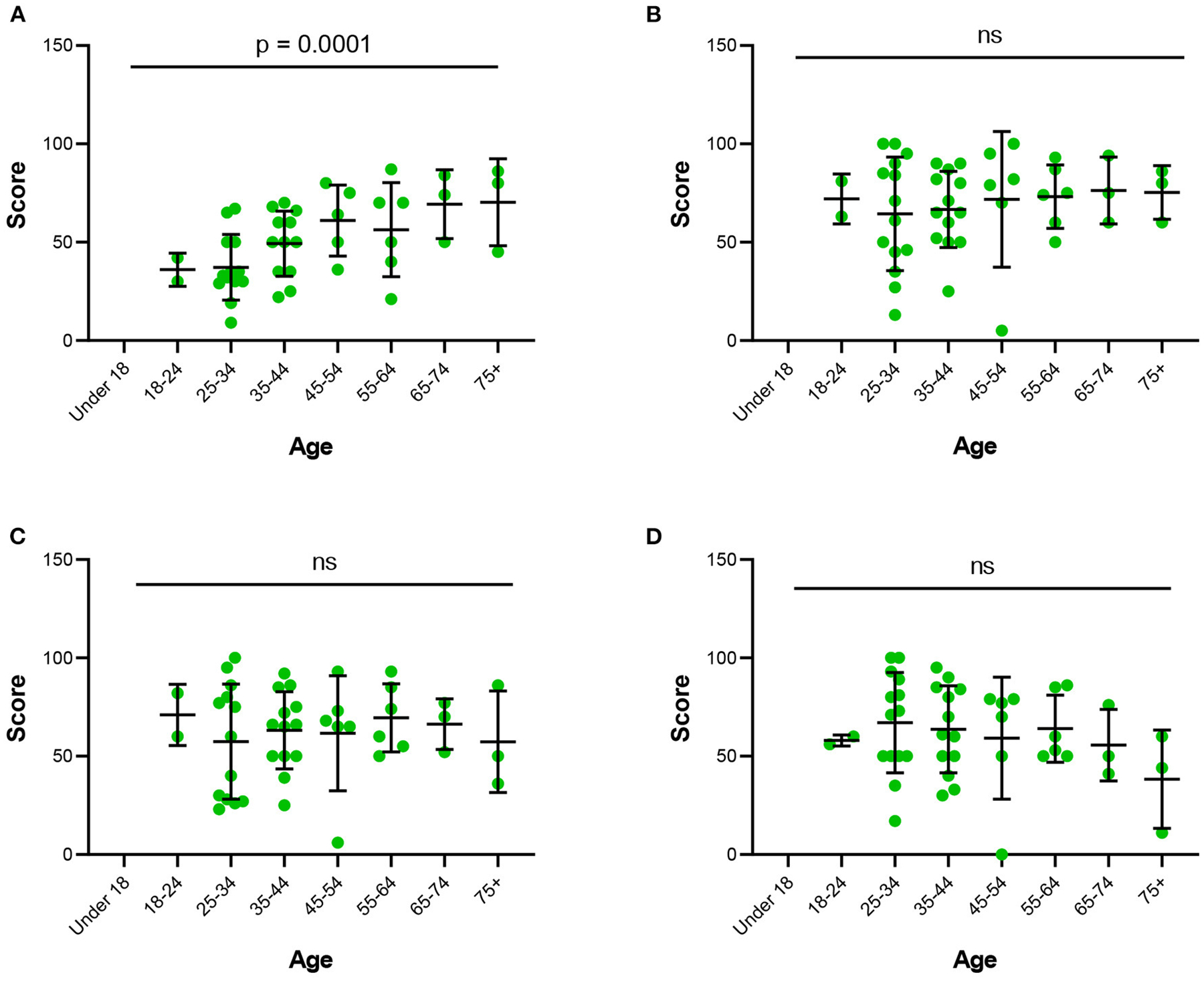
Individual scores from the post-conference survey based on attendee’s age. Individual scores from the post-conference survey addressing **(A)** expectation, **(B)** satisfaction, **(C)** confidence, and **(D)** navigation based on attendee’s age. Error bars represent standard errors of mean. Statistical analysis by Spearman’s rho correlation coefficient **[(A)**: *p* = 000.1, **(B)**: *p* = 0.434, **(C)** *p* = 0.779, **(D)** *p* = 0.199]. ns, not significant represents a *p*-value > 0.05.

Survey responders were asked to use a value scale of 0–100, with 100 representing the highest value, with which to rate the quality of the conference, expectations before the conference, satisfaction of the conference, and confidence in future online conferences. The quality of the conference received an average rating of 68.08 out of a possible 100 points (SD = 22.94). Survey responders within the 18–34 age groups had the lowest average expectations score for conference quality, and those between 45 and 54, and 65+ years of age had the highest (*p* = 0.0001; Figure 4A). Across all age groups, average satisfaction with conference quality remained consistent, with no statistical significance in difference among age groups (*p* = 0.434; Figure 4B). Those between 18 and 24 years of age indicted the highest level of confidence in online conferences and those above 75 years of age indicated the lowest level of confidence (*p* = 0.779; Figure 4C). Additionally, no statistical significance was observed when survey respondents were asked to rate their experience navigating SOCIO (*p* = 0.199; Figure 4D). Those between ages 25–34 were observed to have the lowest calculated mean in satisfaction with interactions, followed by those over age 75.

**FIGURE 5:**
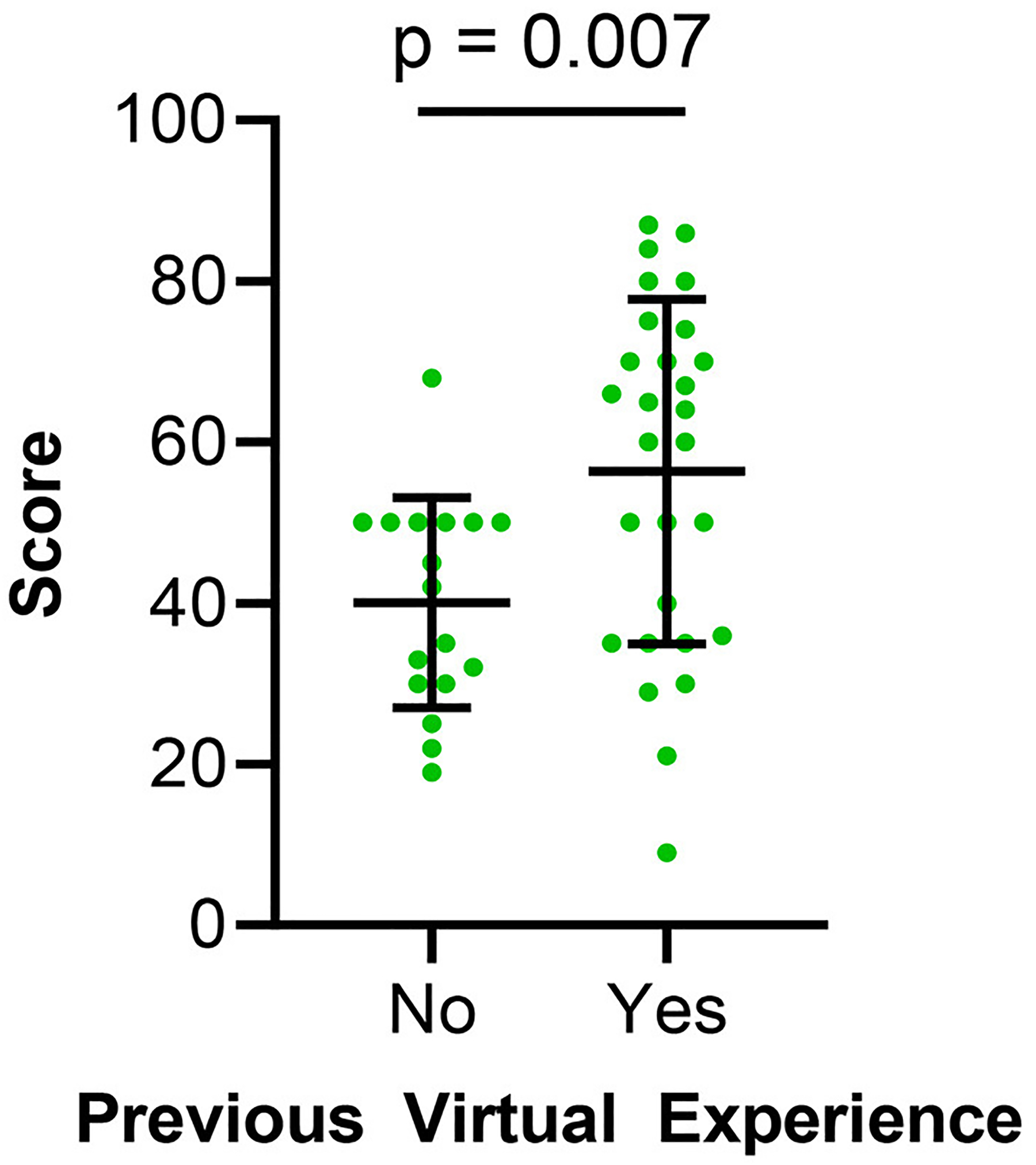
Individual scores from the post-conference survey addressing expectation based on previous virtual conference experience. Error bars represent standard errors of mean.

Responses indicating expectations for the SIPS conference, satisfaction with the SIPS conference [Levene’s test: *p*-value = 0.6863, ANOVA: *p*-value = 0.0438], confidence in virtual conferences overall [Levene’s test: *p*-value = 0.7081, ANOVA: *p*-value = 0.5415], and navigation of the SOCIO platform [Levene’s test: *p*-value = 0.03, ANOVA (not assuming equal variances): *p*-value = 0.9769] were also analyzed in reference to responders’ geographic location. A significant difference was observed in the satisfaction of the SIPS conference (*p*-value: 0.0438) but other calculated mean scores between locations did not show a significant difference. Lastly, responses to these four items were also analyzed in relation to whether an attendee had previously experienced a hybrid/virtual conference or no previous virtual conference experience. Those with no previous virtual experience showed a significantly lower level (*p* = 0.007) in expectations for the SIPS conference compared to those with experience with virtual/hybrid conferences (Figure 5). Satisfaction, confidence, and navigation showed no significant difference between the two groups.

**FIGURE 6:**
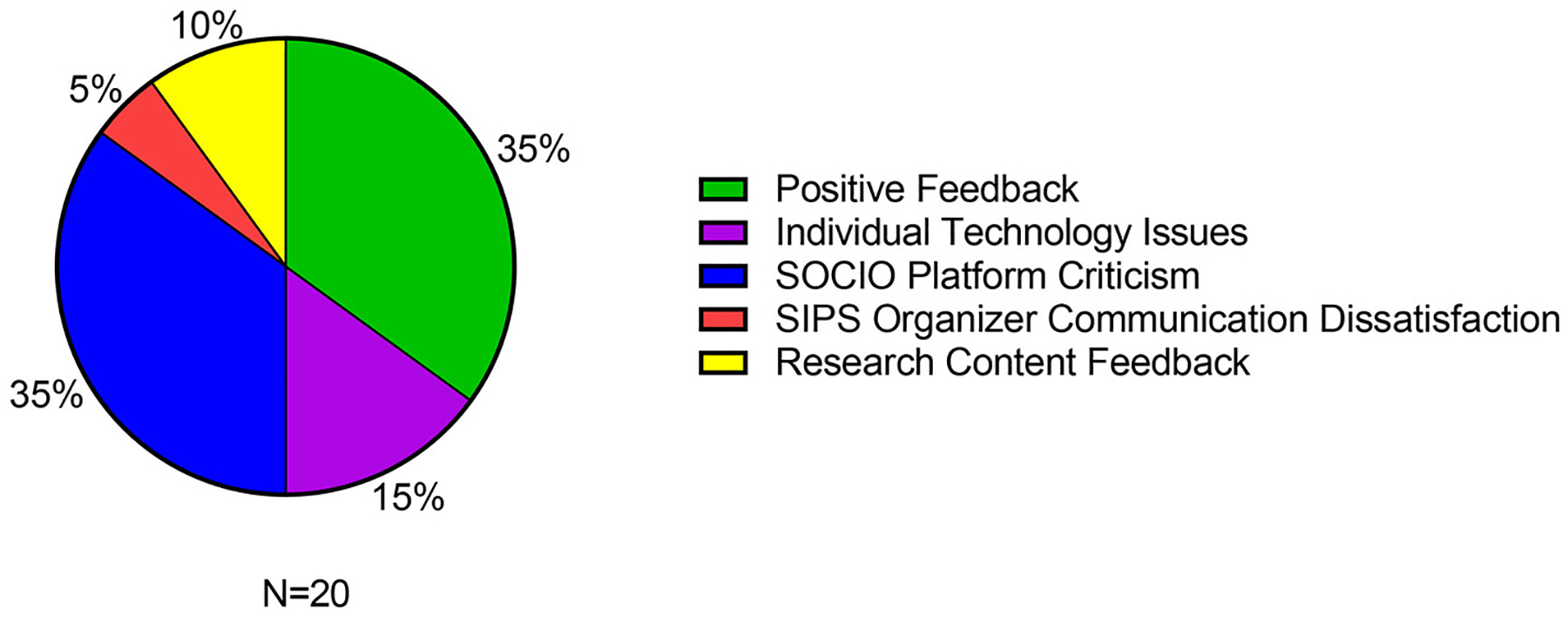
Overall feedback on SIPS 2021 conference based on post-conference survey.

Participants were given a space at the end of the survey to offer additional comments and feedback. Responses from the 17 participants who completed this section, shared elements that can be described as generally positive feedback (*n* = 7), individual technology issues (*n* = 3), criticisms of the SOCIO platform (*n* = 7), dissatisfaction with SIPS organizer communication (*n* = 1), and feedback about the research content of the SIPS conference (*n* = 2) (Figure 6; Supplementary Table 2). These responses from survey participants are a key part of gauging how attendees felt in their own words, in addition to the scores they selected in the items that were presented to them.

## Discussion

Due to the COVID-19 pandemic, the 3rd International Conference of the Society for Interdisciplinary Placebo Studies (SIPS) transitioned to a virtual setting. At that time, few studies provided a quantitative analysis of virtual conferences, leaving a gap in understanding the effect of many features of virtual conferences as well as a lack of evidence with which to develop best practices for the future. With the SOCIO platform and post-conference survey, we were able to collect quantitative and qualitative data with insights into attendance levels, level of engagements, attendee satisfaction, and limitations experienced at the SIPS conference with implications for designing future scientific conferences.

### Attendance level

Total attendance remained fairly consistent across the 3 days with 74% of active users attending all 3 days. The SIPS conference was not the only virtual conference to see a general high retention rates across multi-day conferences (Fulcher et al., 2020; Stamelou et al., 2021; Weiniger and Matot, 2021; Kim et al., 2022) with some reporting an increase in attendance compared to in-person meetings from previous years (Counsell et al., 2020; Fulcher et al., 2020; Stefanoudis et al., 2021; Weiniger and Matot, 2021). However, other conferences held during the COVID-19 pandemic that distributed post-conference surveys did not present an objective assessment of percentage of attendees that could and could not have attended an in-person conference if offered (Ruiz-Barrera et al., 2021; Kim et al., 2022). Over a third of the attendees who completed the SIPS survey were only able to join because the conference was offered virtually. One survey responder stated in reference to the option of a virtual formatted conference in the future: “I am a very old man [….] I need to be very careful regarding this virus. That will be the first thing in line when future conferences come up on the radar.”

To improve accessibility, inclusion, and attendance, research conferences may consider adding a virtual option, creating a hybrid meeting format. Notably, the hybrid format has been explored in conference settings with the ease of restrictions on travel and gatherings. These conferences experienced similar advantages with the majority reporting attendees would like to have virtual options in the future due to reduction of conference cost, attendance flexibility, and reduce carbon footprint (Counsell et al., 2020; Hanaei et al., 2020; Martinelli et al., 2021; Ostler et al., 2021; Sanberg et al., 2021; Chandler et al., 2022; Vartanian, 2022). One suggestion given by Parncutt et al. (2021) discussed the potential for hybrid conferences with multiple “hubs” around the world. Offering the conference experience to more individuals by a virtual option may facilitate increased dissemination of novel findings and spark more cross-continental collaborations, which is especially valuable for emerging scientific fields.

### Level of engagement

Attendees had opportunities to engage in conversation and form connections with other participants within the virtual conference platform using designated networking events, 24/7 available networking rooms, private chat function, chat features during sessions, poster sessions with live video, and Q&A rooms after sessions. Virtual conferences during the COVID-19 pandemic devised different ways to incorporate networking in order to create some semblance of in-person conferences (Veldhuizen et al., 2020; Bhargava et al., 2021; Zaver et al., 2021; Kim et al., 2022). There were conferences that described similar functions to the SIPS Conference virtual platform including private chat functions and chat feature during sessions (Holman et al., 2021; Ruiz-Barrera et al., 2021). Other conferences were unable to incorporate poster sessions due to the limitations in their virtual platform (Bosslet et al., 2020; Ostler et al., 2021). To utilize poster sessions in a hybrid setting, one conference chose to have all poster presenters provide a 5-min pre-recorded talk so the virtual attendees could experience poster sessions online (Chandler et al., 2022), whereas, the SIPS conference offered this option for those who could not make their poster session. Interestingly, due to the lack of networking capability after the conclusion of conference sessions, attendees of one conference created a Google Doc themselves to further network after each session had completed (Bosslet et al., 2020). This suggests attendees place a high level of value on conference networking opportunities. Providing a 24/7 networking option, similar to the SOCIO’s networking rooms, is strongly recommended, especially for international conferences where different time zones need to be considered.

Approximately one fifth of the SIPS conference active users made use of the networking rooms available, with the most unique users on the first conference day. Similarly, both poster sessions experienced a relatively low level of attendance. Further, poster placement appeared to have some effect on level of viewing. Therefore, the format should support equal viewing of all posters. One suggestion is having small icons representing each poster that can be viewed on one screen. When a cursor is hovered over these icons, each poster would expand. This could be a better option in giving a fair chance to all posters. This is especially important if judging of posters is done by the general audience. The majority of SIPS Conference posters included links to external files, which the audience appeared to use. These external files consisted of either an audio or video presentation of the poster through SoundCloud or Vimeo, respectively. These additional tools for engagement gave users the opportunity to participate similarly to activities seen in an in-person conference. However, this benefit would only be applicable if the participants chose to make use of these platform features. Making poster sessions and other organizationally challenging events online requires careful consideration of the usability of specialized features as well as communication with attendees.

### Attendee satisfaction

The post-conference survey allowed participants to not only expand on their answer selections, but also to share thoughts regarding related subjects that were not included in the survey. Four of the survey participants shared frustrations related to the limitations of the Q&A and discussion rooms. Specifically, the rooms’ limited capacity (15 attendees) and the additional step needed to navigate to these rooms, were perceived as barriers to participation. A number of virtual conference features need refining, and these additional comments responses provided valuable perspectives. In future virtual conferences, Q&A and/or discussion rooms may not be necessary if the original presentation room remained open and allowed attendees to join by live stream to ask questions and expand the conversation. These concerns have been seen in other conferences, stating that virtual networking was not the same as in-person networking (Newman et al., 2021; Stamelou et al., 2021). Conferences that utilized Zoom, for example, had the ability to see each attendee face to face with the speaker. Attendees from this conference showed preference for a virtual face-to-face with everyone in the conference (Stamelou et al., 2021). Thus, considering both the attendee feedback from the SIPS conference and feedback from other virtual conferences, ease of direct attendee to attendee interaction (e.g., seeing faces, question asking, mic access, chat box access, etc.) should be prioritized by virtual conference planners.

Comparing various age groups’ level of satisfaction with the quality of the conference and ease of use is imperative to ensuring that virtual conferences remain accessible to all populations. The attendee satisfaction results supported that those over 75 years of age had the lowest level of confidence in virtual conferences and had the most difficulty navigating the virtual platform. While this correlation did not demonstrate statistical significance, it may indicate a technology gap between different age groups (Kim et al., 2022). Even though the SIPS Planning Committee reviewed multiple platforms for ease of use, additional studies need to be conducted on best platforms for multiple generational users. It may also be useful to have an interactive tutorial that users can use to familiarize themselves with the many features of the platform. Tech volunteers, accessible *via* a “help” button, that are assigned to help attendees with general issues could also be helpful supports.

It is also imperative to compare an attendee’s location to satisfaction of a conference, especially with attendees joining in a different time zone. According to the post-conference survey, there was no significance difference seen in satisfaction of the quality of the conference when considering geographic location. However, this was not always the case that had attendees from multiple time zones (Ostler et al., 2021). Creating options for attendees from different time zones to network at any point in the day as well as provide recordings soon after each session may enhance international attendees’ sense of inclusion and promote networking across the globe.

### Privacy concerns

The SOCIO platform provided a feature that recorded user interaction with both the SOCIO website and SOCIO mobile application. This feature records what individual users clicked on, and this data is linked to the individual’s conference-registered name. This feature was essential in data collection and provided insight into how users engaged with not only the conference platform features but with one another. However, having an identifiable record of an individual’s online activity may raise concerns about privacy. Many conferences have not explored privacy concerns that is inherently involved with a virtual conference (Karabacak et al., 2021; Ruiz-Barrera et al., 2021; Kim et al., 2022). Privacy issue could be addressed by ensuring that future conference attendees be made aware of the data that platforms such as SOCIO collects.

### Limitations

The COVID-19 pandemic required a new level of use of existing communication technology such as Zoom, WebEx, and Microsoft Teams. The programs listed existed and were used in business, academia, research, and social settings (Roepke, 2020). The SIPS team worked with the virtual conference platform SOCIO to host the conference. Specific tech issues, such as platform usability, or success of certain conference elements, such as poster sessions or connections, were partially dictated by the unique features available from SOCIO. Overall, the results which reflect the planning, executing, and attending the SIPS annual conference, are potentially limited by the particular technological aspects related to SOCIO’s platform.

Another limitation was the inconsistencies observed in the SOCIO automatic platform data collection, which SIPS Committee members discovered during the quality control (QC) process. Missing data points were noted between different data sheets that had collected the same information, thus the data had to be excluded altogether. Furthermore, attendance information had to be provided directly from the SOCIO team. SOCIO explained that the attendance data had a few glitches and incomplete data through the reports for the SIPS conference due to attendees possibly using VPNs or incognito web browsers. In the future, it is recommended to ask platforms companies what the limitations are in their data collection and whether they have QC processes in place if data is going to be used for analysis purposes.

The survey data collected was informative and provided further insightful on how a sub population of attendees felt about the conference itself and virtual conferences in general. However, this survey was completed by only 11% of the attendees and did not show the full extent of locations attendees were from. Other conferences that provided a post-conference survey had varying attendee response rates ranging from 16 to 89.7% (Veldhuizen et al., 2020; Chan et al., 2021; Holman et al., 2021; Karabacak et al., 2021; Nelson et al., 2021; Stamelou et al., 2021; Wang et al., 2021; Kim et al., 2022; Vinchenzo et al., 2022). This difference could be due to the time lag between the end of the SIPS conference and time the survey was delivered (80 days after the conference). The low response rate limits the validity of these results. A reminder to complete the SIPS post-conference survey as an attempt to increase survey participation resulted in an immediate increase in survey responses. Another method to improve survey participation is to provide the survey after each session, at the end of each day, and or after the concluding remarks of the conference. This method has been used which did see an increase in survey responders compared to the SIPS conference (Stein et al., 2021; Vinchenzo et al., 2022).

### Incorporating in-person meetings

In a poll of 900 Nature magazine readers, 74% believed that post-pandemic, scientific meetings should continue to use a virtual format, or have a virtual component (Remmel, 2021). The support for virtual platforms is echoed in other publications on the merits of virtual conferences (Bosslet et al., 2020; Salomon and Feldman, 2020; Hassell and Hassell, 2021; Stein et al., 2021). Participants of COVID-era virtual scientific or research conferences expressed that they would attend virtual conferences after the pandemic and recommend virtual conferences as an option. The overarching positive sentiment suggests that virtual platforms will become an integral part of the scientific research world.

Furthermore, implementing elements of virtual platforms into on-site conferences has potential for promoting research dissemination. A hybrid model could support larger-scale involvement; the internet’s accessibility opens doors to international participants (Levitis et al., 2021). For example, the SOCIO platform used in the SIPS conference offered multi-language closed captioning; this type of feature would promote inclusivity. Hybrid conferences pose practical challenges, but these difficulties could be resolved with further research.

## Conclusion

The analysis of producing a virtual, scientific conference revealed both benefits and challenges of using virtual format. Further, the results suggest that designing a hybrid model for future conferences may enhance access to these forums as well as accelerate dissemination and collaboration. A high attendance and retention rate over all 3 days of the 3rd Annual SIPS International Conference suggested that the virtual platform provided increased accessibility to a world-wide audience. In comparison to overall attendance, the core conference elements, networking forums, and poster sessions produced moderate levels of attendee engagement. The overall flexibility of the virtual conference also gave attendees more independence in their interaction with the conference, but it may also have detracted from the quality of audience interaction experienced by speakers. Conference metrics and attendee satisfaction results suggest that careful consideration of conference goals and user experience when designing conference-specific virtual features, such as networking rooms, connections, and Q&A facilitation, could improve the efficacy of future virtual conferences. Overall, the data gathered from the 2021 SIPS conference supports that the current form of virtual conferences are effective, but improvements can and should be made. Looking forward, a hybrid model poses an opportunity for supplementing in-person conferences with greater accessibility, flexibility, and optimal dissemination.

## Supplementary Material

Supplementary material

## Figures and Tables

**TABLE 1:** Description of each feature listed on the SOCIO platform used during the SIPS conference.

Feature name	Description
Welcome and overview	The conference “Home Page” with general information about the conference and host institution (UMB).
Sponsors	Displayed each sponsor’s logo and mission with links to their respective website.
Agenda	A detailed program schedule with active links that allowed participants to join sessions directly from this page
Speakers	Listed all speakers with list of associated session(s). A link would direct viewer to the speaker’s biography. Attendees could search for speakers by name.
Poster session I and II	Two individual poster sessions. Posters were visible throughout the entire Conference however authors were assigned a specific session where they were present.
Attendees	Listed all registered attendees which could be searched by name and allowed individuals to tag them as a connection.
Announcements	Displayed announcements pertaining to networking rooms, lectures, and Conference updates.
SIPS website	An active link to the conference website which was separate from the SOCIO platform
Message wall	Attendees could write and respond to comments from other attendees.
Q&A rooms	Attendees and speakers could meet after a session to continue discussions.
Networking rooms	Attendees could meet using live video and audio features.
PS Polling	Rated the posters based on scientific merit as well as visuals and presentation skills.

**TABLE 2:** Attendees’ country of affiliation.

Affiliated country	Number of attendees (*n* = 353)	Percentage (%)
Australia	10	2.8
Brazil	5	1.4
Canada	10	2.8
Denmark	6	1.7
France	1	0.3
Germany	84	23.8
Hong Kong	1	0.3
Ireland	2	0.6
Italy	12	3.4
Netherlands	25	7.1
Norway	1	0.3
Poland	8	2.3
Portugal	1	0.3
South Africa	1	0.3
Spain	4	1.1
Sweden	4	1.1
Switzerland	14	4.0
Taiwan	1	0.3
United Kingdom	6	1.7
United States	157	44.5

## Data Availability

The raw data supporting the conclusions of this article will be made available by the authors, without undue reservation.
