# Supplementary material for: Integration of virtual platforms for enhanced conference experience: Data-based evidence from the Society of Interdisciplinary Placebo Studies 2021 conference
Source: Front Commun (Lausanne). Author manuscript; Available in PMC 2022 Sep 7. (PMC9451137; doi:10.3389/fcomm.2022.857661)
Supplement: Supplementary material [file NIHMS1834211-supplement-Supplementary_material.pdf]

Supplementary Material

1 Supplementary Data

1.1 Post conference 2021 SIPS survey

Disclosure Statement

SIPS Conference. The information from this survey will benefit future participants in advancing future conferences. This survey contains up to 17 questions about the 2021 SIPS conference and is expected to take 5-10 min for completion. Participation is voluntary and all individuals who participate in this survey will be unidentifiable, and any questions that one wish may be left unanswered. Participation is complete once you have answered all desired questions and submitted the survey. You may withdraw from participation at any time while taking the survey by not submitting it. The risk to survey participants may include stress, anxiety, boredom, and inconvenience. For any questions, comments, or concerns, please contact Dr. Chamindi Seneviratne at sips2021_volunteers@lists.umaryland.edu.

Participant Background Information

Please select your age range:

- ○ Under 18
- ○ 18-24
- ○ 25-34
- ○ 35-44
- ○ 45-54
- ○ 55-64
- ○ 65-74
- ○ 75-+
- ○ Prefer not to answer

Please select your current career stage (select all that apply)

- ☐ Student
- ☐ Early Level Career
- ☐ Mid Level Career

☐ Senior Level Career

☐ Other

Please select your academic discipline (select all that apply):

☐ Medicine

☐ Nursing

☐ Pharmacy

☐ Psychology

☐ Social Work

☐ Law

☐ Policy

☐ Other

Please select the geographic region where you were when attending the conference

○ African Continent

○ Asian/Pacific

○ Eastern Europe

○ Latin America

○ Middle East

○ North America

○ South America

○ Western Europe

○ Prefer not to answer

I attended the conference as a (select all that apply):

☐ Attendee

☐ Poster presenter

☐ Plenary speaker

☐ Speaker

☐ Other

Please specify: ________________________________

Select one of the following statements that best describes your intentions about the 2021 SIPS conference, IF the COVID-19 pandemic had not occurred:

- ○ I would only have been able to attend this conference if it were offered on a virtual platform
- ○ I would have been able to the conference if it were held in Baltimore, Maryland

My previous Experience with virtual conferences is best described as:

- ○ This was the first virtual conference I attended
- ○ I have attended a hybrid-style virtual conference
- ○ I have attended an entirely virtual conference
-

Conference Experience

Prior to attending the 2021 SIPS conference, my expectations regarding the quality of a virtual conference experience were:

Extremely low Neither low nor high Extremely High

0 50 100

(place a mark on the scale above)

After Attending the 2021 SIPS conference, I would rate my satisfaction with the quality of the conference as:

Extremely low Neither low nor high Extremely High

0 50 100

(place a mark on the scale above)

After attending the 2021 SIPS conference I would rate my confidence in virtual conferences as:

Extremely low | Neither low nor high | Extremely High

0 | 50 | 100

(place a mark on the scale above)

The following statement best describes my experience navigating SICIO:

Extremely low | Neither low nor high | Extremely High

0 | 50 | 100

(place a mark on the scale above)

Did this deter you from participating in the conference?

- o Yes
- o No
- o Other

Please Specify: ____________________________

The communication provided prior to the conference was:

- o Inadequate
- o Somewhat inadequate
- o Neither adequate nor inadequate
- o Somewhat adequate
- o Adequate
- o Does not apply to me

The communication and the tech support provided prior to, during, and after my presentation was:

- o Inadequate
- o Somewhat inadequate
- o Neither adequate nor inadequate
- o Somewhat adequate

- Adequate
- Does not apply to me
-

The level of attendee interaction I experienced with my presentation was:

Extremely low	Neither low nor high	Extremely High
0	50	100

(place a mark on the scale above)

Was this level of interaction adequate?

- Inadequate
- Adequate
- Other

Please specify: ___________________________

Future Plans

I would attend the next SIPS conference:

- Only if it was entirely in person
- Only if it was entirely virtual
- If it was hybrid (both virtual and in person options available)
- I plan to attend regardless of format
- I do not plan to attend the next SIPS conference

Please select which best characterizes your decision:

- I would plan to attend entirely virtually
- Would plan to attend both virtually and in person
- I would plan to attend entirely in person
- I have not decided

Please select which best characterizes your decision:

- I was dissatisfied with this past conference's quality and/or content
- This conference no longer meets my professional needs
- Reasons unrelated to quality and/or content of this past conference

Please specify: ________________________

Generally, I would attend a research conference in the future if:

- If it was entirely in person only
- If it was entirely virtual only
- If it was hybrid (both virtual and in person options available)
- Other

Please select which best characterizes your decision:

- I would plan to attend entirely virtually
- I would plan to attend both virtually and in person
- I would plan to attend entirely in person
- I have not decided

Please specify: _________________________

Optional Fill in Blank

If you would like to add further comments, concerns, or ideas on how this conference and/or virtual conferences in general can be improved, please do so below:

2 Supplementary Figures and Tables

2.1 Supplementary Table 1. Demographics of participants who completed the post conference survey

Survey Demographics	Total Population n=48	Percentage
Age		
Under 18	0	0.0%
18-24	2	4.2%
25-34	14	29.2%
35-44	13	27.1%
45-54	6	12.5%
55-64	6	12.5%
65-74	3	6.3%
75+	3	6.3%
Prefer not to answer	1	2.1%
Career Stage*		
Student	6	12.5%
Early-Career Investigators	15	31.3%
Mid-Career Investigators	11	22.9%
Senior Investigators	17	35.4%

Other	1	2.1%
Academic Discipline*		
Medicine	15	31.3%
Nursing	1	2.1%
Pharmacy	1	2.1%
Psychology	23	47.9%
Social Work	0	0.0%
Law	1	2.1%
Policy	1	2.1%
Other	12	25.0%
Geographic Region		
Middle East	1	2.1%
North America	21	43.7%
South America	1	2.1%
Western Europe	23	47.9%
Prefer not to answer	1	2.1%
Attended Conference as*		
Attendee	26	54.2%
Poster presenter	8	16.7%

Plenary speaker	3	6.3%
Speaker	19	39.6%
Other	2	4.2%

* Participants had the ability to choose more than one option

2.2 Supplemental Table 2. Attendee quotes from the open-ended post-conference survey. Post conference 2021 SIPS survey.

Positive Feedback	"Thank you so much! I had a very good time during the conference😊"
	"I thought the online platform was excellent, and loved the breakout rooms for discussion."
	"Great conference! I thoroughly enjoyed it."
	"I respect and appreciate the hard work of the leadership team."
	"Thank you for this great conference and very good organization!"
	"The conference organizers did a fantastic job. This was a well-planned and organized international conference. The topic list was extremely well-devised."
	"The quality of the program and speaker, and the organisation were excellent !"
Individual Technology Issues	"When presenting my poster, I was booted out of my room whenever someone tried to join."
	"We lost power in [our area] hence I could not use [SOCIO] to interact for several hours with the conference participants"
	"Needed better training on how to manipulate the the technology."
SOCIO Platform Criticism	"I do not think the socio format was the best option. It did not really encourage exchange and discussion"
	"It was unfortunate that the discussions after the talks were restricted to only 12 or so people, it would be interesting to listen to the questions and discussions"
	"Everyone tried very hard. But the unknown qualities of many details (like showing a slide, ordinarily a trivial issue) were large here. I don't think it's anyone's fault, but the software is new and not perfect."
	"The only thing that was not perfect to me was the restriction to 15 attendees in discussion rooms."
	"I have presented at other virtual conferences where it is posisble to see the audience and interact much more direclty with them. I much prefer such platforms as they promote meangingful interaction far better than the platform used at the SIPS conference."
	"All of the trainings did not prepare me for the fact that once i started my presentation i would not see any comments or controls on my screen."
	"I think a separate room [Q&A]created an unnecessary barrier."

<table>
<tr><td>SIPS Organizer Communication Dissatisfaction</td><td>"When running an international conference it is essential to clearly state a time and timezone for any deadlines, e.g. for abstract submission. This was not done for this conference and it was incredibly frustrating to enter all of my details only to be kicked out of the system for missing the deadline which had not been clearly stated."</td></tr>
<tr><td rowspan="2">Research Content Feedback</td><td>"A general comment that is unrelated to the format is that the conference was very much oriented around the clinical perspective"</td></tr>
<tr><td>"Many of the ... presentations were of high quality but I was unpleasantly surprised by the low quality of [some] talks."</td></tr>
</table>
